# Tuberculosis outbreaks in schools: Experiences from the Western Pacific Region

**DOI:** 10.5365/wpsar.2020.11.3.005

**Published:** 2021-01-26

**Authors:** Kalpeshsinh Rahevar, Tracy Yuen, Kyung Hyun Oh, Seiya Kato, Yuhong Liu, Zhang Lijie, Jingtao Gao, Liang Li, Zi Chen, Cheon Tae Kim, Sarankhuu Amarzaya, Fukushi Morishita, Tauhid Islam

**Affiliations:** a End TB and Leprosy Unit, Division of Programmes of Disease Control, World Health Organization Regional Office for the Western Pacific, Manila, Philippines.; b Research Institute of Tuberculosis, Japan Anti-Tuberculosis Association, Tokyo, Japan.; c National Clinical Center on Tuberculosis, Beijing, China.; d Department of International Cooperation, Innovation Alliance on TB Diagnosis and Treatment, Beijing, China.; e Korean Institute of Tuberculosis, Seoul, Republic of Korea.; f Ministry of Health and National Center for Communicable Disease, Ulaanbaatar, Mongolia.

## Abstract

Reports of tuberculosis (TB) outbreaks among schoolchildren have increased in recent years in countries across the Western Pacific Region. Cases from China, Japan, Mongolia and the Republic of Korea were studied to derive lessons from the challenges and responses to TB outbreaks in schools. Despite differences in the TB burden and outbreak preparedness, the four countries reported similar challenges. These included delayed diagnosis of index cases, lack of experienced health professionals and sustained financial support, and difficulty in responding to intensified media and community attention. Early detection of outbreaks, established resource mobilization networks, coordination among stakeholders and proactive communication were highlights of successful outbreak responses. These principles could be adapted to each context for responses to future TB outbreaks in schools.

Despite continued progress in reducing the burden of tuberculosis (TB) in the World Health Organization (WHO) Western Pacific Region, TB remains a leading cause of death from infectious diseases in the Region. (*1*) The TB burden ranges widely across the Region, from countries in which TB has been eliminated as a public health concern to countries with some of the highest burdens of TB globally.

Reports of TB outbreaks among schoolchildren have been increasing recently. Congregated settings, overcrowded classrooms and various risk profiles among students may contribute to rapid transmission of TB in school settings. Moreover, TB outbreaks in schools and among children attract intense media and community attention and increase scrutiny of TB programmes. To date, there are limited international guidelines on responses to TB outbreaks in schools.

We report a range of experiences in responding to TB outbreaks in schools in the Western Pacific Region in four case studies compiled by WHO collaborating centres (in China, Japan and the Republic of Korea) and by the Ministry of Health, Mongolia. These case studies could inform the responses of countries that have minimal experience in responding to and preventing TB outbreaks in schools.

## Case study: China

### TB burden

In 2019, the incidence rate among children aged 0–14 years was 58 per 100 000 population, representing 1% of all notified cases. (*2*)

### Outbreak definition

Ten or more cases or any TB-related deaths associated epidemiologically with a school during one semester.

### Laws, regulations and TB control and management in schools

Standards for TB prevention and control in schools are detailed in the National TB Plan within the Thirteenth Five-year Plan and in Implementing Standards for Tuberculosis Prevention and Control in Schools and the TB Control Action Plan 2019–2022. The last was issued jointly by nine ministries, including the National Health Commission, to increase the capacity of schools to detect TB cases early and to prevent public health emergencies. TB outbreak investigations are guided by the Expert Consensus on Epidemiological Investigation and Onsite Disposition of TB Outbreaks in Schools. TB case reporting is mandated by the Interim Regulation on Public Health Emergencies.

Contacts are screened for TB by chest X-ray and symptoms and for latent TB infection (LTBI) with the tuberculin skin test (TST; recommended) or an interferon-gamma release assay (IGRA).

### Example outbreak

An outbreak was announced in August 2017 after the identification of cases of active TB at a middle school. During the initial investigation, all students and staff were screened four times, and asymptomatic close contacts and classes in which a high TB incidence was detected were screened at regular intervals. The response was guided by a committee of experts, chaired by the county’s top officials. The school infrastructure was disinfected daily. Counselling (at home visits or by phone) and online study schemes were offered to students. The medical costs incurred by students were fully covered by the county government or by medical insurance. Of 72 students treated (29 for pulmonary TB, 5 for presumptive pulmonary TB and 38 for LTBI), 50 satisfied the criteria for successful treatment and resumed school in November 2017.

## Case study: Japan

### TB burden

In 2019, the incidence rate among children aged 0–14 years was 13 per 100 000 population, representing < 1% of all notified cases. (*2*)

### Definition of outbreak

A single-source case infects > 20 persons in more than two families.

### Laws, regulations and TB control and management in schools

Epidemiological surveys and contact investigations for TB are described in the Infectious Diseases Control Law, which prohibits people with smear-positive results from working until their sputum is negative. The country’s contact investigation guide gives the criteria for extending contact investigation. An outbreak identified by a public health centre must be reported to the Ministry of Health, Labour and Welfare.

Public health centres are primarily responsible for contact investigation. The Infectious Diseases Control Law recommends the establishment of an ad hoc outbreak investigation committee that includes officials from schools, local education committees and public health centres, local laboratory staff, TB experts and representatives of local medical associations. Representatives of parents’ associations are usually not invited to preserve the confidentiality of the index case.

Contacts are screened for TB with a chest X-ray and for LTBI with IGRA.

### Example outbreak

An outbreak investigation was initiated after the diagnosis of TB in a junior high-school student in April 2009. The student experienced intermittent fever, cough and sputum for 6 months and visited several clinics 2 months before diagnosis, but did not receive a chest X-ray. Initial investigation of close contacts resulted in the identification of one case of active TB and two cases of LTBI among family members and a high rate of IGRA positivity among classmates. TB preventive treatment was given to 50% of classmates. Another case of pulmonary TB (with the same variable number of tandem repeat patterns as the index case) was identified in July 2009. The second stage of the investigation was campus-wide, and 15 cases of active TB and 45 cases of LTBI were identified overall.

## Case study: Mongolia

### TB burden

In 2019, the incidence rate among children aged 0–14 years was 428 per 100 000 population, representing 10% of all notified cases. (*2*)

### Definition of outbreak

CDC definition of a higher occurrence of cases than expected in a specific area and/or time. (*3*)

### Laws, regulations and TB control and management in schools

As of 2019, there were no regulations or standards for TB management in schools. A meeting of the Ministry of Health, the Ministry of Education, Culture and Sports, the Ulaanbaatar Governor’s office and occupational inspection agency and others was convened to develop plans for the management of TB in schools after reports of TB outbreaks. A draft guideline on TB outbreak management was planned for approval by the Minister of Health.

In contact investigations, younger students are screened for TB with TST and teachers and older students with a chest X-ray.

### Example outbreak

An outbreak was notified after 60 cases of active TB (3% of 1732 students and staff) were reported at a secondary school between 2015 and 2017. In the initial investigation in April 2017, a high proportion (49% of 889 students) of students had a positive TST. Isoniazid preventive therapy could not be offered because of insufficient stock. The second stage of the school-wide investigation was conducted between May and June by chest X-ray for all staff and students in grades 8–12 and/or TST for those in grades 1–7. Two cases of active TB were detected among 1618 students tested. Three subsequent field investigations were conducted 6 months apart on selected students. The response was coordinated jointly by the Khan-Uul District Health Care Centre, the Tuberculosis Surveillance and Research Department and the Diagnostics Division of the National Centre for Communicable Disease. Follow-up investigations were originally planned every 6 months for 2 years or until no new cases were detected. This was not, however, implemented due to limited financial and human resources.

## Case study: Republic of Korea

### TB burden

In 2019, the incidence rate among children aged 0–14 years was 59 per 100 000 population, representing 1% of notified cases. (*2*)

### Definition of outbreak

More TB cases are detected during contact investigations in congregated settings.

### Laws, regulations and TB control and management in schools

The Infectious Diseases Prevention and Control Act states the legal responsibility of the central and local governments for epidemiological investigation. The Tuberculosis Prevention Act details measures to be taken during outbreaks and for the management of contacts of patients with infectious TB.

Once a school reports a TB case to a health centre, the head of the health centre notifies the provincial TB officials, the Korea Disease Control and Prevention Agency (KCDA) and the electronic TB surveillance system. The health centre organizes an investigation team of a physician and a TB nurse from the health centre, a medical officer from the provincial health department, a member of the Tuberculosis Epidemic Investigation Service at the KCDA, the principal and a health teacher from the school and a focal person from the provincial department of education. The health centre is responsible for conducting field investigations, with administrative support from the provincial health department. The health centre also treats patients, reports the results of contact investigations through the TB surveillance system and briefs parents and students when necessary.

Contact investigations are conducted by screening for TB with a chest X-rays and LTBI with IGRA.

### Example outbreak

An outbreak investigation was initiated after a high-school student with active TB was notified in June 2018,  2 weeks after symptom onset. The initial investigation was conducted among 250 individuals by chest X-ray, and another student was diagnosed with active TB. Of the 63 individuals tested for LTBI, 14 (seven close contacts, seven teachers) tested positive.

## Ethics statements

As routinely available data were used and no personal identifying information was collected, ethical clearance was not required.

## Discussion

The four country case studies show the range of experiences in the Western Pacific Region. The lessons and challenges experienced are summarized in **Table 1**. While the countries faced similar challenges, the causes differed according to the TB burden and the resources available. Some elements of the responses differed from WHO guidance on TB management (e.g. environmental sanitation). A systematic review was therefore conducted to guide interpretation of the case studies to understand evidence on TB outbreaks in schools globally (Supplementary material).

**Table 1:** Key challenges and lessons learnt

Challenge	Lessons and solutions
Delayed TB diagnosis	Empower students to identify and manage their health issues proactively by TB-related health promotion in schools and by reducing stigmatization. Outbreaks could be an opportunity to educate health care professionals and the general public about the persistence of TB in their community. Improve schools’ capacity to detect potential TB cases.
Low LTBI treatment uptake	Educate students, teachers and parents about the importance of LTBI treatment. Offer LTBI treatment at the site of the outbreak. Create a treatment monitoring system to ensure that patients initiate and complete the full course of treatment.
Lack of financial support and human resource capacity	Recruit staff (e.g. nurses, laboratory staff) and resources from neighbouring cities or from outside TB teams. The available resources should be assessed at the start of a field investigation to ensure continuity of services.
Poor coordination among stakeholders	Establish national policies and local plans to coordinate outbreak response. Health and education authorities, at all levels, should support schools in prevention and control activities. Clarify the role of each organization to ensure harmony and complementarity.
Media and community attention interference with outbreak response	Outbreak communications should ensure 1) a clear, early announcement, 2) building and maintaining trust, 3) transparency and 4) understanding by the public. Designate a communications staff to coordinate media requests and to provide regular public briefings. Provide TB information and counselling to parents and students from the beginning of the outbreak.

LTBI, latent tuberculosis infection; TB, tuberculosis

In most outbreaks, the index case was a secondary school or university student. As young adults and older children are likely to have more casual contacts than younger students, the size and complexity of field investigations may depend on the age of the affected students. Some guidelines recommend a “stone-in-the-pond” approach to contact investigation, whereby contacts are prioritized according to their risk of exposure and susceptibility to TB; (*4*-*7*) however, the approach is difficult to implement when the index case is a child.

Delayed diagnosis of TB was reported repeatedly. In countries with a low TB incidence, clinicians may be less aware of the symptoms and misdiagnose cases. Delayed diagnosis may also be due to poor knowledge about TB among students, parents and teachers and to poor health-seeking behaviour by students. In some outbreaks, health promotion material has been distributed to increase awareness of TB and reduce stigmatization. China has developed online modules for students with TB to reduce interruption of schooling, which may reduce their hesitancy to seek care.

As field investigations should begin soon after an outbreak is identified, there is a surge in demand for human resources (e.g. clinicians, nurses) and medical supplies (e.g. test kits, drugs). Mongolia was unable to treat patients with LTBI because of a drug shortage and had to limit the number of follow-up investigations because of financial constraints. Japan reported a lack of paediatricians with experience in TB for screening. In some outbreaks, assistance was obtained by recruiting resources from neighbouring districts or by screening over a longer period (Supplementary material). Guidelines suggest that the resources that will be required should be assessed at the beginning of an investigation, so that staff could be recruited from outside TB teams. (*4*, *7*) Sustained financial and human resources are crucial, as investigations may be prolonged. The Republic of Korea reported that a high turnover of nurses disrupts patient monitoring, and school breaks may interrupt field investigations.

The importance of communication was evident, as TB outbreaks in schools often result in intensified attention from the media and communities. Designating communications personnel to coordinate media requests and to brief the public and the media regularly was reported to be valuable. Certain guidelines (*4*-*7*) state that proactive communication with parents, the school administration, students, health practitioners and the general public is essential. The WHO Outbreak Communications Guidelines detail best practices for effective communication with the public during an outbreak. (*8*)

In terms of cooperation in outbreak response, local public health centres were those primarily responsible, sometimes sharing the burden with school officials and external experts. This is consistent with the guideline of the European Centre for Disease Control and Prevention, which recommends that the core TB management team consult when necessary. (*7*) In the four case studies, policies for managing TB outbreaks in schools were usually covered by the national TB strategy, with no specified contribution from other government bodies. A universal coordination mechanism might be difficult to define, as ministry jurisdictions and public health administrative structures differ among countries.

In resource-limited settings, health care programmes may be supplemented with external resources (e.g. from nongovernmental or faith-based organizations). Therefore, the most effective way of coordinating stakeholders is unclear. As large-scale outbreaks could deplete the available resources, guidelines for outbreak prevention and control are critical in resource-limited settings. There is also limited guidance on integrating outbreak response into the national TB programme. There are therefore gaps in the current knowledge base, particularly in settings most impacted by TB outbreaks in schools.

## Conclusion

The case studies illustrate the challenges and lessons learnt from TB outbreaks in schools across the Western Pacific Region. Despite differences in the TB burden and in outbreak preparedness, the countries faced similar challenges. The key lessons include the importance of early outbreak detection to prevent delayed TB diagnosis, establishment of resource mobilization networks to meet the demands for specialized clinicians and supplies for TB  screening and treatment, coordination of stakeholders in non-health sectors (e.g. education) and proactive  communications. Countries could adapt the principals to their context when developing a protocol for the prevention and control of TB outbreaks in schools.
